# The Gulliver syndrome: a conceptual framework to address therapeutic inertia in patients with borderline cardiovascular risk profiles

**DOI:** 10.3389/fcvm.2025.1652447

**Published:** 2025-08-14

**Authors:** José Francisco López-Gil, José Abellán-Huerta, José Abellán-Alemán

**Affiliations:** 1 School of Medicine, Universidad Espíritu Santo, Samborondón, Ecuador; 2 Vicerrectoría de Investigación y Postgrado, Universidad de Los Lagos, Osorno, Chile; 3 Servicio de Cardiología, Hospital General Universitario Santa Lucía, Cartagena, Spain; 4 Sociedad Murciana de Hipertensión Arterial y Riesgo Cardiovascular, Cátedra de Riesgo Cardiovascular, Universidad Católica de Murcia, Murcia, Spain

**Keywords:** obesity, hypertension, diabetes mellitus, cholesterol, biomarkers

## Abstract

We propose a novel clinical construct, the “Gulliver syndrome”, to describe the scenario in which multiple, mildly elevated cardiovascular risk factors (CVRFs) coexist within an individual and together result in a significantly heightened overall risk of cardiovascular disease (CVD). This accumulation of small deviations, often dismissed in clinical practice, can exert a synergistic impact on vascular health. Our aim is to formalize this underrecognized phenotype, which falls outside traditional diagnostic entities such as the metabolic syndrome, and to provide a framework that enables early recognition and management. We outline proposed diagnostic criteria, contrast this syndrome with related constructs, support its clinical relevance with emerging literature, and present a representative case. Ultimately, we advocate for this framework as a tool to overcome therapeutic inertia and encourage proactive, multifactorial interventions in primary and preventive care.

## Introduction

A recurring theme in preventive cardiology is the tendency to underestimate the cumulative impact of multiple subclinical or borderline risk factor elevations. While clinical guidelines provide thresholds that delineate normal from pathological, the pathophysiology of atherosclerotic cardiovascular disease (ASCVD) is a continuum. Even modest elevations in blood pressure, lipids, or glycemia can interact in complex ways to accelerate endothelial dysfunction, inflammation, and plaque formation (1).

The “Gulliver syndrome” is named after Jonathan Swift's fictional character Lemuel Gulliver, who, though a giant among the Lilliputians, finds himself incapacitated by countless tiny ropes. Analogously, many patients are immobilized by multiple minor aberrations in cardiovascular risk factors (CVRFs), each one clinically unalarming, yet jointly capable of creating a high-risk internal milieu. These patients often remain untreated or undertreated due to the absence of any single alarming metric, a phenomenon termed “therapeutic inertia” (2).

We argue that current frameworks, such as the metabolic syndrome, fall short in capturing this phenotype. While the metabolic syndrome focuses on obesity and insulin resistance, the Gulliver syndrome emphasizes the cumulative burden of borderline CVRFs independent of overt obesity or glucose dysregulation. Moreover, recent literature has challenged the predictive value of some metabolic syndrome criteria, such as low high-density lipoprotein (HDL) cholesterol, as independent causal factors (3–5). This phenomenon is particularly relevant in patients with borderline or mildly elevated cardiovascular risk profiles, who often remain below treatment thresholds despite accumulating multiple non-optimal markers.

### Proposed definition and diagnostic criteria

The Gulliver syndrome is characterized by the simultaneous presence of at least four mildly abnormal CVRFs, each falling within a “borderline” range but together elevating total cardiovascular risk.

Mandatory criteria (all four must be present):

- Waist circumference (WC): 90–101 cm in men or 80–87 cm in women [borderline abdominal adiposity; measured in a clinical setting under standardized conditions, values between optimal and diagnostic thresholds (≥102 cm in men, ≥88 cm in women), associated with increased cardiometabolic risk] (6–8).
- Systolic blood pressure (SBP): 120–139 mmHg and/or diastolic blood pressure (DBP): 80–89 mmHg [borderline blood pressure; measured in a clinical setting under standardized conditions, below diagnostic hypertension thresholds (≥140/90 mmHg), but above optimal levels] (9, 10).
- Fasting plasma glucose (FPG): 100–125 mg/dL [borderline glycemia; measured after ≥8 hours of fasting, below the diagnostic threshold for diabetes (≥126 mg/dL), but consistent with impaired fasting glucose] (11).
- Non-HDL cholesterol: 130–189 mg/dL [borderline-to-high range; associated with increased atherogenic burden without reaching the threshold for very high-risk dyslipidemia (≥190 mg/dL)] (8, 12–14).

Additional aggravating factors (non-essential but contributory):

- Sedentary lifestyle.
- Smoking.
- Alcohol consumption.
- Psychosocial stress.
- Family history of premature cardiovascular disease (CVD).
- Unhealthy diet.
- Short or disrupted sleep.

**Figure 1:**
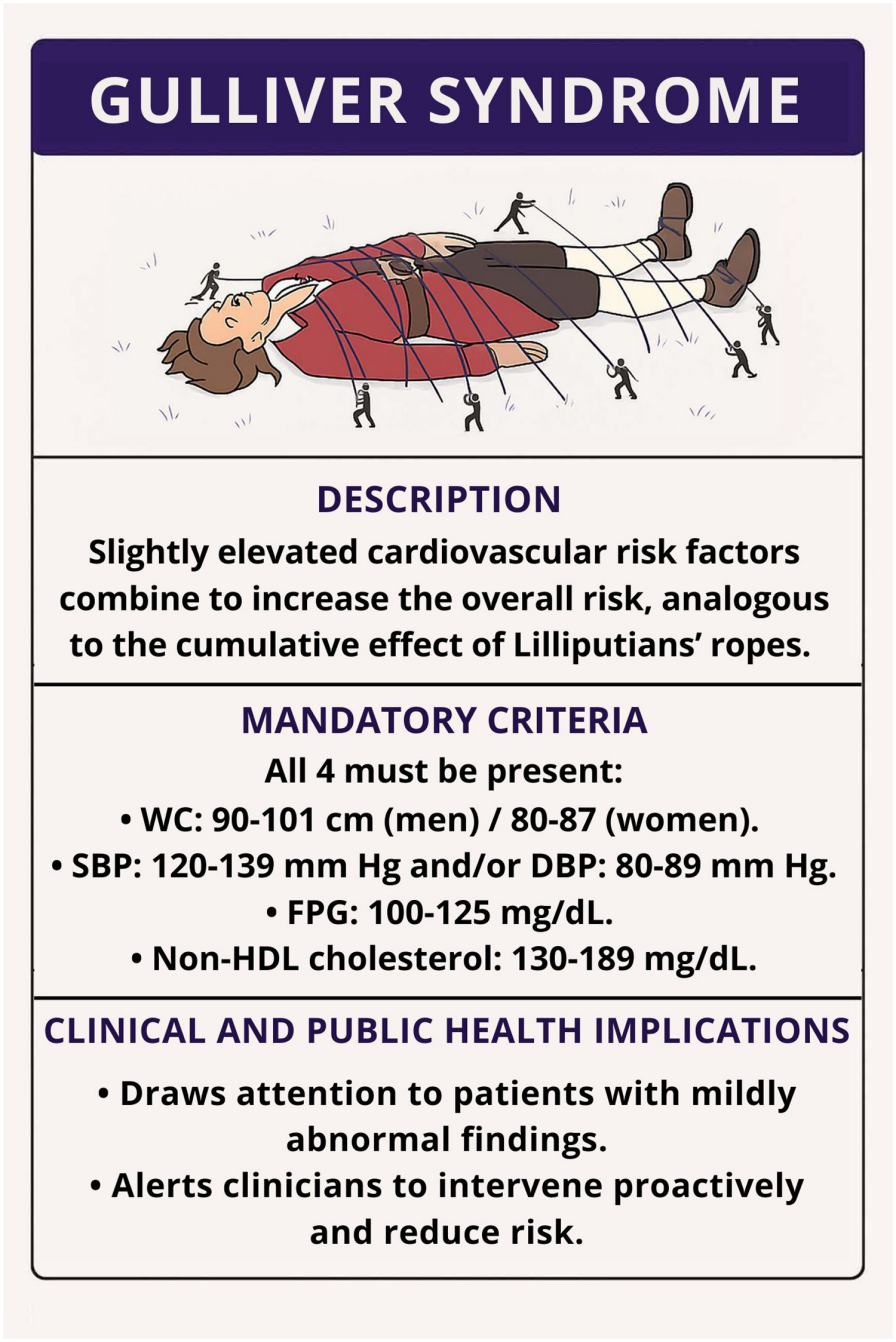
Graphical representation of the Gulliver syndrome. A patient's overall cardiovascular risk increases when multiple mildly elevated risk factors coexist. Each rope represents a single, subclinical abnormality (waist circumference, blood pressure, glucose, or non-high-density lipoprotein colesterol) that collectively restrain health, analogous to the ropes of the Lilliputians. DBP, diastolic blood pressure; FPG, Fasting plasma glucose; HDL, high-density lipoprotein; SBP, systolic blood pressure; WC, waist circumference.

Although not formally included in the diagnostic core, these factors may act as amplifiers of cardiovascular risk and are therefore relevant in the Gulliver syndrome framework. These criteria are grounded in evidence linking each variable with elevated cardiovascular morbidity and mortality (even at sub-threshold levels) when present in combination (15–17). Figure 1 illustrates the proposed concept of Gulliver syndrome, highlighting the convergence of borderline CVRFs and their cumulative impact on overall cardiovascular risk.

#### Case illustration

Mr. A, a 52-year-old male software engineer, presents for an annual check-up. He is asymptomatic, non-smoking, and has no prior diagnosis of diabetes or hypertension. He reports minimal physical activity and frequent job-related stress. On physical examination and laboratory evaluation, the following findings were noted:

- WC: 96 cm.
- Blood pressure: 128/84 mmHg.
- FPG: 106 mg/dl.
- Total cholesterol: 220 mg/dl; HDL cholesterol: 48 mg/dl; Non-HDL cholesterol: 172 mg/dl.

Despite each parameter being only mildly abnormal, his calculated 10-year Systematic Coronary Risk Evaluation 2 (SCORE2) cardiovascular risk places him in the moderate-to-high category. Yet no therapeutic intervention is initiated beyond generic lifestyle advice. This reflects a missed opportunity for early intervention and risk modification.

#### Theoretical basis and pathophysiological rationale

Evidence from longitudinal cohort studies demonstrates that cardiovascular risk accumulates progressively with incremental elevations in risk factors, even below diagnostic cut-offs. For instance, Vasan et al. (18) showed that individuals with prehypertension and borderline cholesterol levels had a significantly increased lifetime risk of coronary artery disease.

Furthermore, the concept of “residual risk” (i.e., the risk that remains even after managing a primary risk factor) underscores the need for multifactorial control. The Gulliver syndrome, by identifying patients with diffuse low-grade risk, may help target this residual burden earlier.

The pathophysiology supports this notion: even small elevations in glucose and blood pressure can impair endothelial function, increase arterial stiffness, and enhance oxidative stress. Subclinical inflammation (a hallmark of early atherogenesis) is fueled by such chronic metabolic perturbations (19–21).

#### Implications for clinical practice and policy

Therapeutic inertia, defined as the failure to initiate or intensify therapy when indicated, has been widely documented in hypertension, type 2 diabetes, and dyslipidemia (22–24). It is particularly prevalent in patients with borderline abnormalities, where no single value crosses the treatment threshold.

By defining and naming Gulliver syndrome, we provide a conceptual tool to prompt clinician awareness. This syndrome encourages the use of composite risk scores (e.g., SCORE2, ASCVD Risk Estimator) and favors the early use of lifestyle modification and, when appropriate, pharmacologic intervention. The polypill strategy is especially relevant for such patients (25).

Moreover, as cardiovascular prevention shifts toward precision medicine, identifying subtle but meaningful phenotypes like Gulliver syndrome could enhance individualized care pathways and inform public health strategies (Figure 1).

The American Heart Association's Life's Essential 8 framework, which includes metrics such as diet quality, physical activity, sleep health, and nicotine exposure (26), offers a holistic approach to cardiovascular health that complements our conceptualization of Gulliver syndrome. A recent meta-analysis has shown that a large proportion of adults score poorly across several Life's Essential 8 components (27), especially in domains such as diet and sleep, reinforcing the prevalence of diffuse risk accumulation. Recognizing individuals with poor composite metrics could support earlier identification of Gulliver syndrome candidates and reduce therapeutic inertia. Thus, while the Life's Essential 8 provides a population-level assessment tool, the Gulliver syndrome framework adds clinical granularity by identifying individuals who, despite lacking severe abnormalities, may benefit from targeted preventive intervention.

## Conclusion

The Gulliver syndrome encapsulates a common but under-recognized clinical reality: the aggregation of mild, subclinical CVRFs that, if unaddressed, lead to significant morbidity and mortality. Formal recognition of this construct may reduce therapeutic inertia, enhance preventive strategies, and improve long-term outcomes. We call for empirical studies to quantify its prevalence and impact, and advocate for its inclusion in clinical education and decision-support tools.
