# Correction: The Gulliver syndrome: a conceptual framework to address therapeutic inertia in patients with borderline cardiovascular risk profiles

**DOI:** 10.3389/fcvm.2025.1696969

**Published:** 2025-11-06

**Authors:** José Francisco López-Gil, José Abellán-Huerta, José Abellán-Alemán

**Affiliations:** 1 School of Medicine, Universidad Espíritu Santo, Samborondón, Ecuador; 2 Vicerrectoría de Investigación y Postgrado, Universidad de Los Lagos, Osorno, Chile; 3 Servicio de Cardiología, Hospital General Universitario Santa Lucía, Cartagena, Spain; 4 Sociedad Murciana de Hipertensión Arterial y Riesgo Cardiovascular, Cátedra de Riesgo Cardiovascular, Universidad Católica de Murcia, Murcia, Spain

**Keywords:** obesity, hypertension, diabetes mellitus, cholesterol, biomarkers

Reference 10 for McEvoy et al. was erroneously written as “McEvoy JW, McCarthy CP, Bruno RM, Brouwers S, Canavan MD, Ceconi C, et al. 2024 ESC Guidelines for the management of elevated blood pressure and hypertension. Eur Heart J. (2024) 45(38):3912–4018. doi: 10.1093/eurheartj/ehae178It”.

This reference should be:

“Jones DW, Ferdinand KC, Taler SJ, Johnson HM, Shimbo D, Abdalla M, et al. 2025 AHA/ACC/AANP/AAPA/ABC/ACCP/ACPM/AGS/AMA/ASPC/NMA/PCNA/SGIM Guideline for the Prevention, Detection, Evaluation and Management of High Blood Pressure in Adults: A Report of the American College of Cardiology/American Heart Association Joint Committee on Clinical Practice Guidelines. Circulation. (2025) 152(11). doi: 10.1161/CIR.0000000000001356”.

There were errors in the Description and Mandatory criteria sections of Figure 1.

The text previously read:

Description

- “…analogous to the cumulative effect of the Lilliputians’ rapes.”

Major cardiovascular risk factors

- WC: 90–102♂ cm/80–88♀ cm.
- SBP: 120–139 mm Hg; DBP: 80–89 mm Hg.
- FBG: 100–125 mg/dL.

The corrected text is below:

Description

- “…analogous to the cumulative effect of the Lilliputians’ ropes.”

Mandatory criteria

All 4 must be present:

- WC: 90–101 cm (men)/80–87 cm (women).
- SBP: 120–139 mm Hg and/or DBP: 80–89 mm Hg.
- FPG: 100–125 mg/dL.

There was a mistake in the caption of Figure 1 as published: “FBG, Fasting blood glucose” should be “FPG, Fasting plasma glucose.”

The updated Figure 1 and its caption appear below.

**Figure 1:**
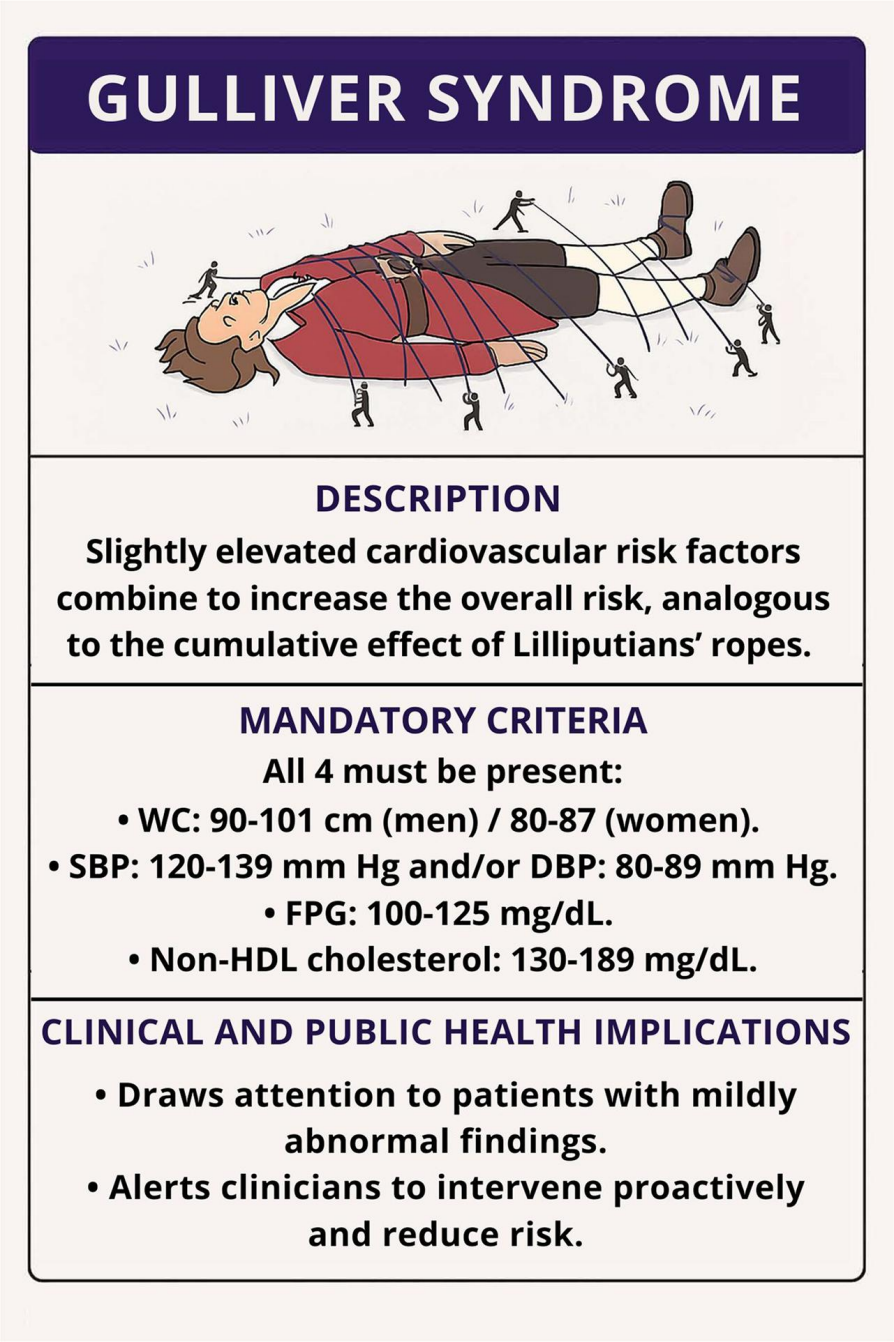
Graphical representation of the Gulliver syndrome. A patient's overall cardiovascular risk increases when multiple mildly elevated risk factors coexist. Each rope represents a single, subclinical abnormality (waist circumference, blood pressure, glucose, or non-high-density lipoprotein colesterol) that collectively restrain health, analogous to the ropes of the Lilliputians. DBP, diastolic blood pressure; FPG, Fasting plasma glucose; HDL, high-density lipoprotein; SBP, systolic blood pressure; WC, waist circumference.

A correction has been made to the values and terms in the sections “Proposed definition and diagnostic criteria” and “Case illustration”.

The text previously read:

Proposed definition and diagnostic criteria

- Systolic blood pressure (SBP): 121–139 mmHg and/or diastolic blood pressure (DBP): 81–89 mmHg.
- Fasting plasma glucose (FPG): 101–125 mg/dL.

Case illustration

HDL-C.

The corrected text is below:

Proposed definition and diagnostic criteria

- Systolic blood pressure (SBP): 120–139 mmHg and/or diastolic blood pressure (DBP): 80–89 mmHg.
- Fasting plasma glucose (FPG): 100–125 mg/dL.

Case illustration

- HDL cholesterol.

The original version of this article has been updated.

